# O-Glycosyltransferase Gene *BnaC09.OGT* Involved in Regulation of Unsaturated Fatty Acid Biosynthesis for Enhancing Osmotic Stress Tolerance in *Brassica napus* L.

**DOI:** 10.3390/plants13141964

**Published:** 2024-07-18

**Authors:** Cui Liu, Qingyang Li, Shan Peng, Li He, Ruihua Lin, Jiahui Zhang, Peng Cui, Hongbo Liu

**Affiliations:** The College of Advanced Agricultural Sciences, Zhejiang A & F University, Hangzhou 311300, China; lcui2021@163.com (C.L.); 18155507167@163.com (Q.L.); 15565691285@163.com (S.P.); 19851335721@163.com (L.H.); lrh_0207@163.com (R.L.); z1277236091@163.com (J.Z.)

**Keywords:** *Brassica napus* L., O-glycosyltransferase, drought stress, unsaturated fatty acid, gene function

## Abstract

Osmotic stress is a major threaten to the growth and yield stability of *Brassica napus*. Post-translational modification with O-linked β-N-acetylglucosamine (O-GlcNAc) is ubiquitous in plants, and participates in a variety of signal transduction and metabolic regulation. However, studies on the role of O-GlcNAc transferase (OGT) in osmotic stress tolerance of plants are limited. In previous study, a O-glycosyltransferase, named BnaC09.OGT, was identified from the *B. napus* variety ‘Zhongshuang 11’ by yeast one hybrid with promoter of *BnaA01.GPAT9*. It was found that BnaC09.OGT localized in both nucleus and cytoplasm. The spatiotemporal expression pattern of *BnaC09.OGT* exhibited tissue specificity in developmental seed, especially in 15 days after pollination. In view of osmotic stress inducing, the *BnaC09.OGT* overexpression and knockout transgenic lines were constructed for biological function study. Phenotypic analysis of *BnaC09.OGT* overexpression seedlings demonstrated that *BnaC09.OGT* could enhance osmotic stress tolerance than WT and knockout lines in euphylla stage under 15% PEG6000 treatment after 7 days. In addition, compared with WT and knockout lines, overexpression of *BnaC09.OGT* had significantly higher activities of antioxidant enzymes (SOD and POD), higher content of soluble saccharide, and while significantly less content of malondialdehyde, proline and anthocyanidin under 15% PEG6000 treatment after 7 days. On the other hand, the unsaturated fatty acid content of *BnaC09.OGT* overexpression was significantly higher than that of WT and knockout lines, so it is speculated that the BnaC09.OGT could increase unsaturated fatty acid biosynthesis for osmotic stress tolerance by promoting the expression of *BnaA01.GPAT9* in glycerolipid biosynthesis. In summary, the above results revealed that the function of BnaC09.OGT provides new insight for the analysis of the pathway of O-glycosylation in regulating osmotic stress tolerance in *B. napus*.

## 1. Introduction

*Brassica napus* L. is one of the most important oil crops in the world. The entire growth and development period is sensitive to water deficiency [1,2]. Osmotic stress inhibits metabolic processes and thereby reduces biomass accumulation and disrupts seed formation, and ultimately resulting in reduced yield [3,4]. Osmotic stress has a major limiting impact on its production and yield stability, and accounts for a 30% yield loss at least in *B. napus* every year [3,5]. To ensure crop production and quality under limited conditions, developing resistant varieties is an efficient strategy [6,7].

Glycosyltransferases (GTs), which catalyze glycosyl groups to various receptor molecules, including proteins, carbohydrates, lipids, hormones, and flavonoids [8,9,10], are widely present in organisms. This catalytic process alters the biological ctivity, stability, solubility, and transport characteristics of the receptor molecules [11,12], playing a crucial role in maintaining intracellular homeostasis. Additionally, the glycosylated products generated have numerous biological functions. Based on the similarity of amino acid sequences of glycosyltransferases, the specificity of catalytic substrates, and the stereochemical structure of the catalytic products, the glycosyltransferases are classified into 135 families according to Carbohydrate-Active Enzymes Database [13]. The largest and most extensively studied branch in plants is GT1. There is a subclass known as UDP-glucosyltransferases (UGTs) due to their utilization of UDPG as the glycosyl donor [14].

It is found that glycosylation modifications of certain small molecule metabolites are related to the regulation of plant growth and development and stress resistance [15]. In *Arabidopsis thaliana*, glycosyltransferases UGT79B2 and UGT79B3 were involved in glycosylating anthocyanins, and mutations in these enzymes impacted the accumulation of anthocyanins, which reducing the tolerance to adverse conditions such as salt, drought, and cold stress [16]. After overexpression of *UGT75D1* in *A*. *thaliana*, there is an enhanced tolerance to osmotic stress during seed germination, leading to increased germination rates [17]. Research reported that the UDP-glucosyltransferase GSA1 in rice exhibited glycosyltransferase activity towards flavonoid substrates, mediating auxin transport by affecting flavonoid content, ultimately regulating rice grain size [18].

Based on the types of glycosidic bonds, protein glycosylation can be classified into four types of glycosyltransferases: OGT, NGT, CGT, and SGT, with OGT being the predominant type in plants [19]. O-glycosylation typically occurs on the oxygen atoms of serine or threonine side chains [20]. O-glycosylation interacts with other PTMs (various types of post-translational modifications) [21,22]. O-GlcNAcylation levels are regulated by O-GlcNAc transferase (OGT) and O-GlcNAcase (OGA). The dynamic and reversible modification of glycosylation, serving as a crucial regulatory factor in cellular processes such as signal transduction, transcription, translation [21,22,23], and the involvement of substrate proteins in multiple cellular signaling pathways [22].

It is also found that glycosylation reactions played a crucial role in plant growth and development, secondary metabolism, hormone balance, and responsed to biotic and abiotic stresses [15,24]. There is limited research on O-GlcNAcylation in plants, and the role of O-GlcNAc modification in plant responses to osmotic stress remains to be investigated. Hereby, a O-glycosyltransferase was identified in *B. napus*, which is classified as OGT and belongs to the GT90 family. Furthermore, the transgenic lines with overexpression and knockout of *BnaC09.OGT* were constructed for biological function study. It was speculated that *BnaC09.OGT* may enhance osmotic stress tolerance by improving the ROS scavenging capacity and increasing the proportion of unsaturated fatty acids in *B. napus*, providing new insights into the pathways by which O-GlcNAc modification regulates plant osmotic stress tolerance.

## 2. Results

### 2.1. Identification of BnaC09.OGT and Phylogenetic Analysis

**Figure 1:**
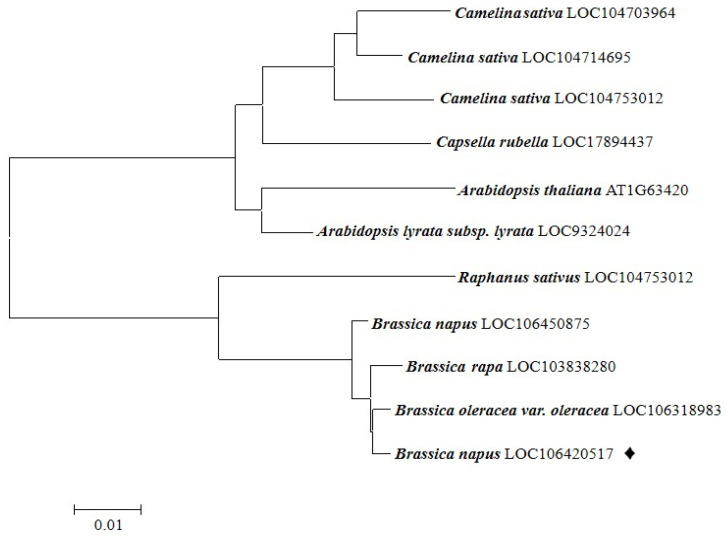
Phylogenetic tree of homologous sequences of BnaC09.OGT protein.

The *BnaC09.OGT* gene was cloned from the cDNA of *B. nupus* by reverse transcript PCR. The sequence length of *BnaC09.OGT* was 1743 bp (Supplementary Figure S1). It was deduced that the coding sequence of the *BnaC09.OGT* gene encodes 580 amino acids, and the protein encoded with a molecular weight of 66.9 kDa by SnapGene software. The conserved domain characteristic of the GT90 glycosyltransferase family was found in protein sequence. Phylogenetic analysis showed that the closest evolutionary relatives of BnaC09.OGT, arranged from nearest to furthest, were: *Brassica oleracea*, *Brassica rapa*, *Raphanus sativus*, *Arabidopsis lyrata*, *Arabidopsis thaliana*, *Capsella rubella*, *Camelina sativa* (Figure 1).

### 2.2. Subcellular Localization of BnaC09.OGT

**Figure 2:**
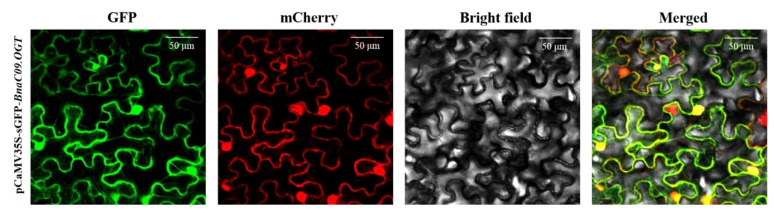
Subcellular localization of BnaC09.OGT. GFP: GFP channel; mCherry: red fluorescence in the nucleus; Bright field: light microscopy image; Merged: merged image of the GFP and Bright channels.

To determine the subcellular localization of the BnaC09.OGT protein, a recombination plasmid of pCAMBIA1305.1-*BnaC09.OGT*-GFP was constructed. Then, the BnaC09.OGT-GFP fusion protein was transiently expressed in *Nicotiana. tabacum* leaves. The result showed that the green fluorescence signal of fusion protein was located in both the nucleus and cytoplasm by laser confocal microscope (Figure 2).

### 2.3. Tissue-Specific Expression Analysis of BnaC09.OGT

**Figure 3:**
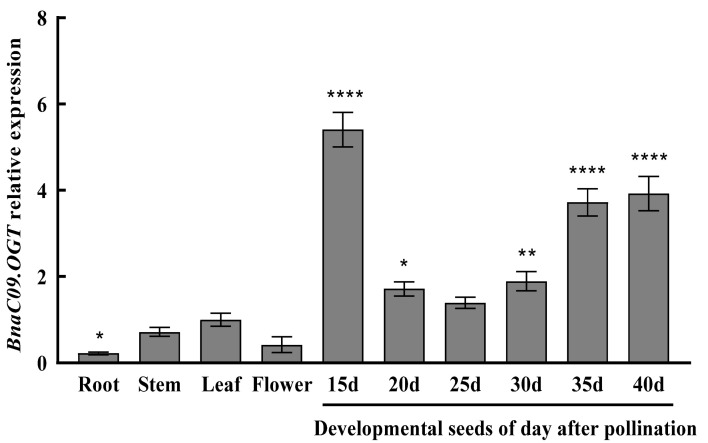
Relative expression pattern of *BnaC09.OGT* in different tissues of ‘Zhongshuang 11’ *: *p* < 0.05; **: *p* < 0.1; ****: *p* < 0.001.

The expression patterns of *BnaC09.OGT* were analyzed by RT-qPCR in root, stem, leaf, flower, and seed of different developmental stages (15, 20, 25, 30, 35, 40 days after pollination). The result indicated that the relative expression of *BnaC09.OGT* was higher in the seed at 15 days after pollination, reaching 5.4 times than that in the leaf (Figure 3).

### 2.4. Analysis of the Expression Pattern of the BnaC09.OGT Gene under Osmotic Stress and Exogenous Hormone Treatments

**Figure 4:**
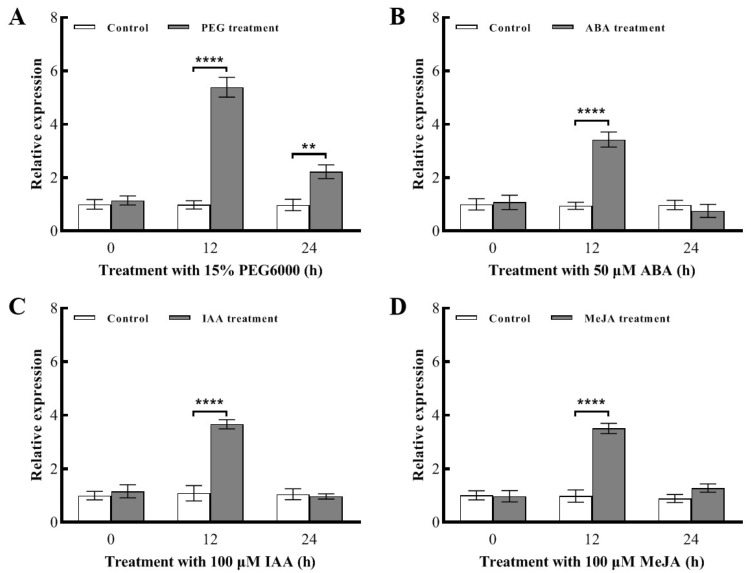
Analysis of the expression pattern of *BnaC09.OGT* gene in leaves under osmotic stress and exogenous hormone treatment. (**A**): 15% PEG6000 treatment; (**B**): 50 μmol/L ABA treatment; (**C**): 100 μmol/L IAA treatment; (**D**): 100 μmol/L MeJA treatment. **: *p* < 0.1; ****: *p* < 0.001.

To ascertain the response to osmotic stress and hormone treatments of the *BnaC09.OGT* gene, the three-leaf stage of ‘Zhongshuang 11’ was subjected to osmotic stress and external application of ABA, IAA, and MeJA, using untreated wild types as controls. Measured the realative expression levels of the *BnaC09.OGT* in leaves under different treatment by RT-qPCR. It was found that the expression of *BnaC09.OGT* peaked at 12 h under osmotic stress, reaching 5.5 times than that of the control (Figure 4A). With ABA IAA, and MeJA treatments, the expression of *BnaC09.OGT* also peaked at 12 h after treatment, reaching 3.6, 3.4, and 3.6 times than that of the control, respectively (Figure 4B–D).

### 2.5. Construction and Identification of Transformants

Two recombinant plasmid, pCAMBIA1305.1-35S-*BnaC09.OGT*-NOS and PKSE401-TRNA were constructed (Supplementary Figure S2), and used to *Agrobacterium tumefaciens*-mediated for hypocotyl genetic transformation (Supplementary Figure S3). 17 positive transgenic lines of *BnaC09.OGT* overexpression was identified by specific PCR (Supplementary Figure S4). Then, T_1_ generation obtained by self-pollination in these lines, and used to screen single-insert transgenic lines with hygromycin (30 μg/mL). Three high expression and single insertion lines, *BnaC09.OGT*-OE1, OE10 and OE14 (Supplementary Figure S5), were selected for further experiments. On the other hand, two knockout lines of *BnaC09.OGT* were identified by high-throughput sequencing and created self-pollination T_1_ generation for subsequent experiments (Supplementary Figure S6).

### 2.6. Characterization of BnaC09.OGT Transformants with Osmotic Stress

**Figure 5:**
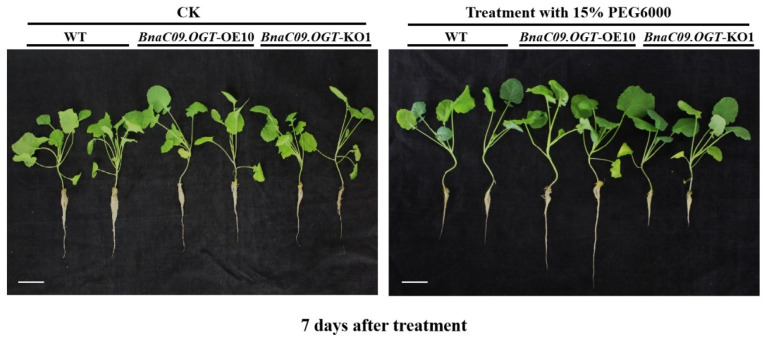
The seedling phenotype of independent *BnaC09.OGT*-OE10 and *BnaC09.OGT*-KO1 lines under osmotic stress. CK: Non-treatment with 15% PEG6000; WT: ‘Zhongshuang11’ variety; *BnaC09.OGT*-OE10: pCAMBIA1305.1-35S-*BnaC09.OGT*-NOS transformant line in ‘Zhongshuang 11’ genetic background; *BnaC09.OGT*-KO1: *BnaC09.OGT* knockout line in the ‘Zhongshuang 11’ genetic background. Bar = 5 cm.

For osmotic stress, the seedling at four euphylla stage of *BnaC09.OGT*-OE1/OE10/OE14 and *BnaC09.OGT*-KO1/KO2 T_1_ generation independent lines were transferred into 1/5 strength Hoagland’s solution with 15% (*w*/*v*) PEG6000 (−0.3 Mpa). Plant phenotypic and related physiological parameters were measured after 7 days osmotic stress treatment. During the early vegetative growth stage, the biomass of shoot was positively correlated with osmotic stress resistance. After treatment, the leaves of the *BnaC09.OGT*-OE independent lines were larger than those of the WT and *BnaC09.OGT*-KO independent lines, and the root length also showed the same exhibition in these transformants and WT (Figure 5). The number of euphylla in overexpression seedlings was significantly more than that of *BnaC09.OGT*-KO and WT lines after 14 days osmotic stress treatment (Table 1).

### 2.7. Physiological Responses to Osmotic Stress

**Figure 6:**
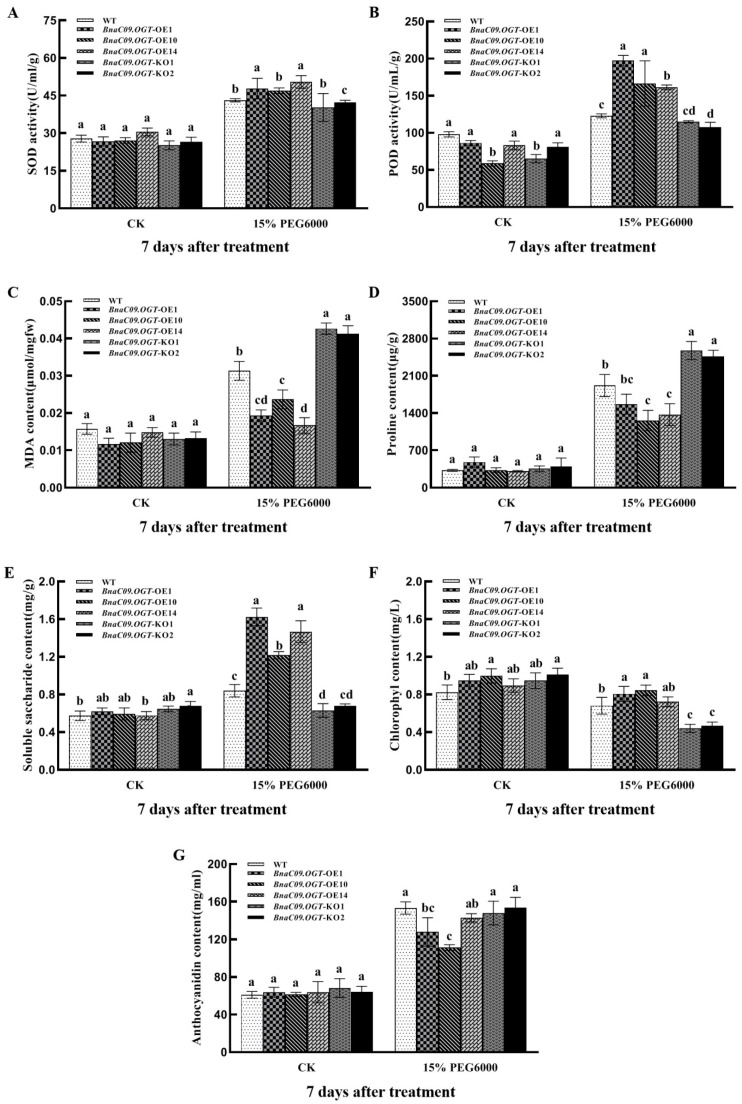
Analysis of physiological indices in *B*. *napus* seedlings with overexpression and knockout of *BnaC09.OGT* under osmotic stress. (**A**): SOD activity; (**B**): POD activity; (**C**): MDA content; (**D**): Proline content; (**E**): Soluble saccharide content; (**F**): Chlorophyll content; (**G**): Anthocyanin content; CK: untreated group: cultivate with 1/5 Hoagland solution; WT: ‘Zhongshuang 11’; *BnaC09.OGT*-OE1/OE10/OE14: independent lines of transformant with pCAMBIA1305.1-35S-*BnaC09.OGT*-NOS in ‘Zhongshuang 11’ genetic background; *BnaC09.OGT*-KO1/KO2: independent lines of transformant with *BnaC09.OGT* knockout in ‘Zhongshuang 11’ genetic background. Values are means of three biological replicates followed by the same letter did not significantly difference at *p* < 0.05.

**Figure 7:**
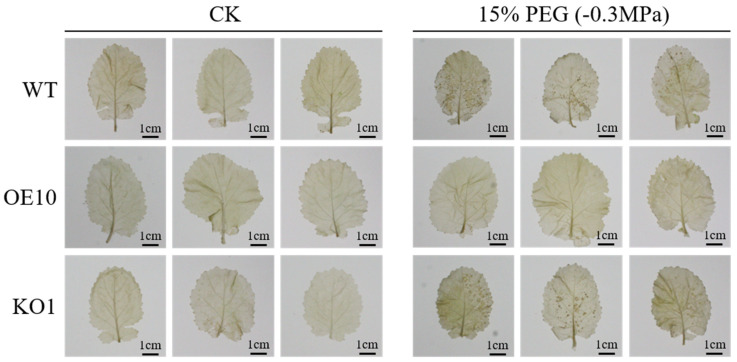
Superoxide radical levels in the leaves of *BnaC09.OGT* transfromants. CK: untreated group: cultivate with 1/5 Hoagland solution; WT: ‘Zhongshuang 11’; *BnaC09.OGT*-OE10: pCAMBIA1305.1-35S-*BnaC09.OGT*-NOS transformant line in ‘Zhongshuang 11’ genetic background; *BnaC09.OGT*-KO1: *BnaC09.OGT* knockout line in ‘Zhongshuang 11’ genetic background.

The enzymatic activity of SOD and POD were calculated to further investigate the effect of osmotic stress in transformants. The results showed that *BnaC09.OGT*-OE independent lines had a significantly higher SOD and POD enzyme activity than *BnaC09.OGT*-KO independent lines and WT (Figure 6A,B). Meanwhile, more MDA accumulation was found in the *BnaC09.OGT*-OE independent lines (Figure 6C). In addition, proline levels increased in all lines after 7 days drought treatment, but the *BnaC09.OGT*-OE lines had lower proline content than WT, while the *BnaC09.OGT*-KO lines had higher content (Figure 6D). Soluble saccharide content was significantly increased in the *BnaC09.OGT*-OE lines compared to WT, and significantly decreased in the *BnaC09.OGT*-KO lines (Figure 6E). The chlorophyll content in all lines decreased after osmotic stress, with the greatest reduction in the *BnaC09.OGT*-KO lines and the least reduction in the *BnaC09.OGT*-OE lines (Figure 6F). The anthocyanin content increased in the WT, *BnaC09.OGT*-OE, and *BnaC09.OGT*-KO lines, while the rate of increase in the *BnaC09.OGT*-OE lines was significantly lower than WT and *BnaC09.OGT*-KO lines (Figure 6G). In addition, the intensity of NBT (Nitro Blue Tetrazolium) staining usually reflects the accumulation of superoxide anions in cells. It was found that the *BnaC09.OGT*-OE lines showed the lightest staining under 7 days osmotic stress, which indicated the least accumulation of reactive oxygen species, while the *BnaC09.OGT*-KO lines showed the darkest staining, indicating more accumulation of reactive oxygen species (Figure 7).

### 2.8. Fatty Acid Component of Transformants under Osmotic Stress

There was no significant difference in the ratio of total unsaturated fatty acids to total saturated fatty acids among WT, *BnaC09.OGT*-OE and *BnaC09.OGT*-KO under osmotic stress (Table 2). However, the results exhibited significant difference in the ratio after 7 days osmotic stress, the content of total unsaturated fatty acids decreased in *BnaC09.OGT*-KO, while increased in the *BnaC09.OGT*-OE lines (Table 2).

## 3. Discussion

The O-glycosyltransferase SECRET AGENT (SEC) showed a high alignment of catalytic active sites in plants, which indicated it was a conserved evolution of the OGT gene [25]. *BnaC09.OGT* was localized in the nucleus and cytoplasm, with potential involvement in the modification of nuclear-localized proteins and soluble proteins [25,26]. The AtUGT71C5 could glycosylate ABA to deactivate it. The mutant of *AtUGT71C5* geneshowed that glycosylation levels of ABA were reduced, resulting in increased levels of active ABA, which enhances the tolerance of osmotic stress [27]. In addition, JA was involved in regulating stomatal closure under drought conditions [28]. Studies have shown that auxins are related to plant drought tolerance. Overexpression of *DgIAA21* reduced the drought tolerance of transgenic *Arabidopsis* [29]. The expression of the *BnaC09.OGT* gene was significantly induced by osmotic stress, and its expression was also significantly increased under exogenous treatments with hormones, such as ABA, MeJA, and IAA. These results suggested that the involvement of osmotic stress tolerance by *BnaC09.OGT* may be related to the regulation of plant hormone levels. It is speculated that these hormones could be glycosylated to involving the osmotic signal pathway.

Furthermore, it was found that the enhanced osmotic stress tolerance of the *BnaC09.OGT* overexpression lines may be due to an increased capacity for ROS scavenging and a higher proportion of unsaturated fatty acids in the plants. The antioxidant defense system is one of the abiotic stress response mechanisms. There is a positive correlation between antioxidant enzyme activity and abiotic stress resistance in plant. Under osmotic stress, reactive oxygen species accumulated in the cell membranes could oxidize the unsaturated fatty acids on the glycerol backbone of the membranes, leading to increased membrane permeability, with the level of stress increased, lipid peroxidation intensifies, damaging membrane proteins and lipids, ultimately impairing the normal physiological function of the cell membranes [30,31]. After osmotic stress, the enzymatic activity of SOD, POD significantly increased in the *BnaC09.OGT*-OE lines. Nitro Blue Tetrazolium (NBT) staining results also showed that the *BnaC09.OGT*-OE lines exhibited reduced reactive oxygen species levels compared to the control under osmotic stress. The *BnaC09.OGT* overexpression lines may enhance plant osmotic stress tolerance by increasing antioxidant enzyme activity and reducing ROS levels. The results of decreased MDA, proline, anthocyanin content, and less reduction in chlorophyll content indicated that the oxidative stress level of *BnaC09.OGT* overexpression lines is lower after osmotic stress, thereby enhancing the resistance of plants. After osmotic stress treatment, the content of unsaturated fatty acids in the *BnaC09.OGT*-OE lines was significantly higher than that of the wild type and *BnaC09.OGT*-KO lines. In addition, phosphatidic acid and phosphatidyl choline, which can affect fatty acid composition, were also presumed to be the substrates of glycosyltransferase for involving the phosphatidic acid signaling pathway and glycolipid synthesis through glycosylation. Thereby, phosphatidic acid and phosphatidyl choline may be an important role in maintaining the integrity of cell membrane structures in plants and ultimately affecting plant osmotic stress tolerance.

## 4. Materials and Methods

### 4.1. Plant Materials

The *B. napus* variety ‘Zhongshuang 11’ was used as the plantlet material in the experiments. The hypocotyl was used to genetic transformation as the explant.

### 4.2. BnaC09.OGT Clone and Phylogenetic Analysis

The coding sequences of *BnaC09.OGT* were amplified using the High-Fidelity Enzyme PrimeSTAR Kit (Takara, Japan) with the specific primers listed in Supplementary Table S1. *BnaC09.OGT* sequences of different species were obtained by BLAST (https://blast.ncbi.nlm.nih.gov/Blast.cgi, 5 November 2023), multiple sequence alignment was performed using DNAMAN. Sequence alignment was performed using Clustal W, followed by the construction of a phylogenetic tree using the neighbor-joining (NJ) method with 1000 bootstraps.

### 4.3. Subcellular Localization

Homologous recombination primers were designed using the vector pCAMBIA1305.1-35S-GFP (modified from pCAMBIA1305.1 with a GFP reporter gene by our laboratory) and the *BnaC09.OGT* coding sequence (Supplementary Table S1). The leaf epidermal cells of *N. tabacum* were transiently infected via *A. tumefaciens*-mediated transformation, with GFP fluorescence signals observed after 36 h of cultivation by confocal microscope [32].

### 4.4. Tissue-Specific Expression of BnaC09.OGT

Total RNA of roots, stems, leaves, flowers, and seeds at different developmental stages (15, 20, 25, 30, 35, 40 days post-pollination) in ‘Zhongshuang 11’ was extracted using a TransZol Up Plus RNA Kit (Transgene, Shenzhen, China) and detected using a NanoDrop™ One (Thermo Fisher, Waltham, MA, USA). The cDNA was synthesized using Hifair^®^ 1st Strand cDNA Synthesis SuperMix for qPCR (Yeasen, Shanghai, China). Relative expression of *BnaC09.OGT* in different tissues were determined by CFX Connect™ Optics Module (Bio-Rad, Hercules, CA, USA) with three biological replications. The 20 μL RT-qPCR contained 10 μL of 2 × Hieff qPCR SYBR Green Master Mix (Yeasen, Shanghai, China), 0.004 nM forward primer, 0.004 nM reverse primer, and 9.2 μL of cDNA. The reaction program was initiated by predenaturation at 95 °C for 5 min, and this step was followed by 40 cycles of denaturation (95 °C for 10 s) and annealing (55 °C for 30 s). The reference gene was ubiquitin-conjugating enzyme 9 (accession no. XM_013800933) which was used to normalize the expression levels of *BnaC09.OGT* [33]. Expression results of RT-qPCR were calculated according to the 2^−ΔΔCt^ analysis method [34].

### 4.5. Analysis of the Expression Pattern of the BnaC09.OGT Gene under Osmotic Stress and Various Exogenous Hormone Treatments

To determine the expression pattern of the *BnaC09.OGT* gene under osmotic stress and various plant hormone treatments, the seeds of ‘Zhongshuang 11’ was grown in 1/5 Hoagland solution until the three-leaf stage. The plants were cultured at a temperature of 24 °C with a photoperiod of 16 h/8 h (light/dark), the light intensity is 300–500 μmol m^−2^ s^−1^, and the relative humidity is 60%. The seedlings were subjected to drought treatment using 15% PEG6000 (−0.3 Mpa) replacing the hydroponic solution. Exogenous hormones were applied by spraying solutions of 100 μmol/L IAA, 50 μmol/L ABA, and 100 μmol/L MeJA on the surface of the seedling leaves. Samples were taken from the leaves at 0, 12 and 24 h after treatment, and the relative expression levels of the *BnaC09.OGT* were measured using RT-qPCR, with 12 seedlings sampled per treatment and repeated three times.

### 4.6. Construction and Identification of Transformants

Restriction enzymes *Nco*I and *Mlu*I were used to identified the pCAMBIA1305.1-35S-*BnaC09.OGT*-NOS plasmid, which was constructed through homologous recombination with the sequences listed in Supplementary Table S1. The recombinant plasmid was then transferred into GV3101. Transgenic seedlings were obtained using *B. napus* variety ‘Zhongshuang 11’ as the recipient through hypocotyl transformation [35]. The T_0_ generation transgenic seedlings were screened by PCR, and those positive seedlings were transplanted to the field until T_1_ generation seeds were harvested. The harvested T_1_ seeds were sterilized, sown on 1/2MS solid medium containing 30 µg/mL Hyg, and cultured for 7 days. A chi-square test was used to verify the ratio of resistant to non-resistant seedlings. The single insertion site of the transformant was calculated to 3:1 ratio. Two CRISPR target sites were designed using the website http://crispr.hzau.edu.cn/cgi-bin/CRISPR2/CRISPR (accessed on 30 June 2024). Primers for amplifying the target sites were designed and synthesized. Using the PGTR plasmid as a template, amplification was performed, and the PCR products were incorporated into the final CRISPR expression vector PKSE401-TRNA through homologous recombination. The recombinant plasmid was then transferred into GV3101, with *B. napus* variety ‘Zhongshuang 11’ served as the recipient, and gene-edited seedlings were obtained through hypocotyl transformation.

### 4.7. Characterization and Physiological Responses to Osmotic Stress of BnaC09.OGT Transformants

Select T_1_ generation of transgenic lines seeds for the experiment, with the *B. napus* variety ‘Zhongshuang 11’ served as the WT (wild type). The transgenic lines include BnaC09.OGT-OE1/10/14: the independent lines of pCAMBIA1305.1-35S-BnaC09.OGT-NOS transgenic lines in ‘Zhongshuang 11’ genetic background; and BnaC09.OGT-KO1/KO2: the independent lines of transformant with BnaC09.OGT knockout in ‘Zhongshuang 11’ genetic background. After vernalization, seeds were sown in germination boxes and grown until the cotyledons unfolded and the hypocotyl elongated, then transferred to 1/5 Hoagland solution for cultivation. When seedlings reached the stage of having four fully unfolded true leaves, similarly grown seedlings were transferred into 15% (*w*/*v*) PEG6000 solution prepared with 1/5 Hoagland solution to simulate a −0.3 MPa osmotic stress condition. After 7 days treatment, plant morphology was observed and related physiological parameters were measured.

### 4.8. Fatty Acid Component Analysis

To investigate the effects of overexpression and knockout of *BnaC09.OGT* on lipid synthesis in *B. napus* leaves under normal growth conditions and osmotic stress, 0.1 g of leave samples were analyzed using an Agilent 7890B gas chromatograph (Agilent, Santa Clara, CA, USA). The fatty acids were then methylated and analyzed by gas chromatography to assess the fatty acid composition of each sample. The seed fatty acid composition was determined on the basis of peak areas. Means were analyzed for variance using least significant difference tests at the *p* < 0.05 level of significance.

## 5. Conclusions

The BnaC09.OGT, located in both the nucleus and cytoplasm, as a key factor to response the osmotic stress and hormones such as ABA, IAA MeJA. The *BnaC09.OGT* overexpression can enhance the osmotic stress tolerance in *B. napus*, which by improving ROS scavenging capacity (though SOD and POD), increasing the proportion of unsaturated fatty acids, and reducing oxidative stress level. These results indicated that *BnaC09.OGT* could promoting the downstream gene expression to affect the glycerolipid biosynthesis. The *BnaC09.OGT* gene can be used as a candidate gene for abiotic stress genetic breeding improvement in *B. napus*.

## Figures and Tables

**Table 1:** Statistics on the number of euphylla in wild type, independent *BnaC09.OGT*-OE and *BnaC09.OGT*-KO lines under osmotic stress.

Lines	Before Treatment	14 Days after Treatment
WT	4.0 ± 0.6	5.2 ± 0.7
*BnaC09.OGT*-OE1	4.2 ± 0.7	6.0 ± 0.6 *
*BnaC09.OGT*-OE10	4.0 ± 0.6	6.5 ± 0.9 ****
*BnaC09.OGT*-OE14	3.8 ± 0.6	6.3 ± 0.7 ***
*BnaC09.OGT*-KO1	3.9 ± 0.3	4.8 ± 0.6
*BnaC09.OGT*-KO2	3.8 ± 0.4	4.9 ± 0.5

Note: Values (*n* = 12) are means ± SE, *: *p* < 0.05, ***: *p* < 0.01, ****: *p* < 0.001. WT: ‘Zhongshuang 11’; *BnaC09.OGT*-OE1/OE10/OE14: pCAMBIA1305.1-35S-*BnaC09.OGT*-NOS transformant lines in the ‘Zhongshuang 11’; *BnaC09.OGT*-KO1/KO2: *BnaC09.OGT* knockout lines in the ‘Zhongshuang 11’.

**Table 2:** The proportion of unsaturated fatty acids in leaves of wild type, independent *BnaC09.OGT*-OE and *BnaC09.OGT*-KO lines under osmotic stress with 15% PEG6000.

Lines	Before Treatment	7 Days after Treatment
WT	79.6 ± 0.5 a	77.3 ± 0.3 b
*BnaC09.OGT*-OE1	79.7 ± 1.3 a	82.2 ± 0.5 a
*BnaC09.OGT*-OE10	79.3 ± 0.7 a	81.5 ± 1.1 a
*BnaC09.OGT*-OE14	77.4 ± 1.0 a	81.9 ± 2.6 a
*BnaC09.OGT*-KO1	79.6 ± 0.5 a	71.2 ± 3.1 c
*BnaC09.OGT*-KO2	79.2 ± 0.5 a	74.4 ± 0.7 c

Note: Values (*n* = 3) are means ± SE, the same letter did not significantly difference at *p* < 0.05. WT: Untransformed ‘Zhongshuang 11’; *BnaC09.OGT*-OE1/OE10/OE14: pCAMBIA1305.1-35S-*BnaC09.OGT*-NOS transformant lines in the ‘Zhongshuang 11’; *BnaC09.OGT*-KO1/KO2: *BnaC09.OGT* knockout lines in the ‘Zhongshuang 11’.

## Data Availability

The original contributions presented in the study are included in the article and Supplementary Material, further inquiries can be directed to the corresponding author.

## Supplementary Materials

The following supporting information can be downloaded at: https://www.mdpi.com/article/10.3390/plants13141964/s1, Figure S1 Amplified of *BnaC09.OGT* gene by reverse transcript PCR. Figure S2 The validation of the recombinant plasmid pCAMBIA1305.1-35S-*BnaC09.OGT*-NOS by colony PCR in *E. coli* DH5α. Figure S3 Genetic transformation of hypocotyls with plasmid pCAMBIA1305.1-35S-*BnaC09.OGT*-NOS in the ‘Zhongshuang 11’. Figure S4 Identification of pCAMBIA1305.1-35S-*BnaC09.OGT*-NOS independent T_0_ transgenic lines by PCR. Figure S5 Relative expression level of pCAMBIA1305.1-35S-*BnaC09.OGT*-NOS independent T_0_ transgenic lines. Figure S6 Sequence characteristics of mutation sites in T_0_ generation *BnaC09.OGT* edited lines. Table S1: Information of primer and gene editing target sequences.
