# Supplementary material for: O-Glycosyltransferase Gene BnaC09.OGT Involved in Regulation of Unsaturated Fatty Acid Biosynthesis for Enhancing Osmotic Stress Tolerance in Brassica napus L
Source: Plants (Basel). 2024 Jul 18;13(14):1964. doi: 10.3390/plants13141964 (PMC11280806; doi:10.3390/plants13141964)
Supplement: Supplementary file 1 [file plants-13-01964-s001.zip › plants-3063365-SI.pdf]

Supplementary Table S1 Information of primer and gene editing target sequences

Gene	Primer Sequence5'-3'	Product length	Application
BnaC09.OGT	FP: TCCTTAGCCTCCAAAGATTCC	1851 bp	Gene clone
	RP: CTAACGTAATCCGATAAATAAAAGC		
	FP: GACAGCCCAGATCAACTAGTATGACATCTCTCTTCTCTAAACCTCG	1779 bp	Subcellular localization
	RP: CCTTGCTCACCATGGATCCTTGAACGTTTTTCCAGTAAGCATG		
	FP: CGAGAATTCAAATGTAGCAGATC	73 bp	qRT-PCR for transformants
	RP: CACTGACCATCCATACCCTTC		
	FP: GGGGACTCTTGACCATGGATGACATCTCTCTTCTCTAAACCTCG	1779 bp	Overexpression vector construction
	RP: AATTCACACGTGACGCGTTTATTGAACGTTTTTCCAGTAAGC		
	FP: CCATCAAATTTGCTGTTGAATGGC	683 bp	Identification for transformants
	RP: GACACCGCGCGCGATAATTTATC		
	CTAGAATCACCGCAGAAAAC	-	Target site for gene editing
	ACTGGTACAGGTGCGTGAAC	-	
BnaUBC9	FP: GCATCTGCCTCGACATCTTGA	68 bp	Actin for qRT-PCR
	RP: CGATAGCAGCACCTTGGAGATA		

Note: the restriction enzyme sites are underlined; FP: Forward primer, RP: Reverse primer.

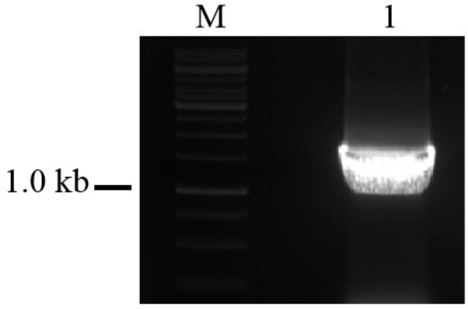

Supplementary Figure S1 Amplified of *BnaC09.OGT* gene by reverse transcript PCR. M: 1 kb DNA ladder; 1: the fragment of *BnaC09.OGT* gene.

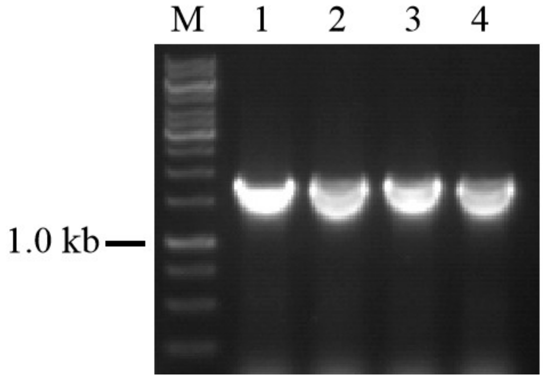

Supplementary Figure S2 The validation of the recombinant plasmid pCAMBIA1305.1-35S-*BnaC09.OGT*-NOS by colony PCR in *E. coli* DH5α. M: 1 kb DNA ladder; 1-4: independent colony with pCAMBIA1305.1-35S-*BnaC09.OGT* -NOS.

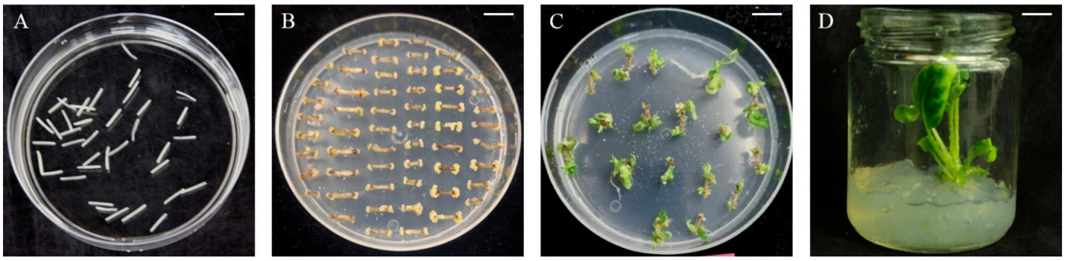

Supplementary Figure S3 Genetic transformation of hypocotyls with plasmid pCAMBIA1305.1-35S-*BnaC09.OGT*-NOS in the 'Zhongshuang 11'. A: hypocotyl; B: callus; C: induced shoots; D: transgenic plantlet. Scale bar =1 cm

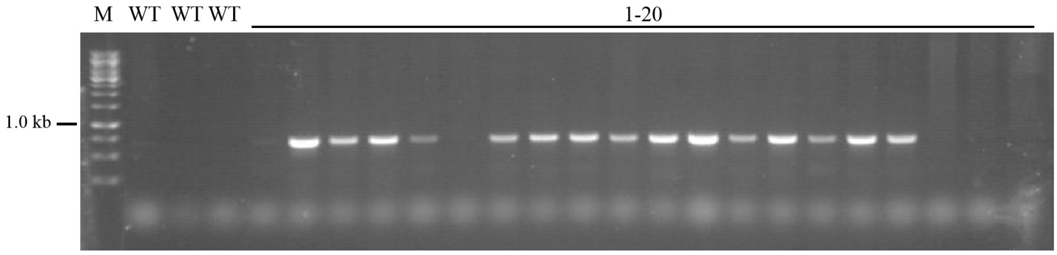

Supplementary Figure S4 Identification of pCAMBIA1305.1-35S-*BnaC09.OGT*-NOS independent T_0 transgenic lines by PCR. The target fragment of specific PCR was 683 bp. M: 1 kb DNA ladder; 1-5 and 7-17: positive plants; 6, 18-20: negative plants; WT: 'Zhongshuang 11'.

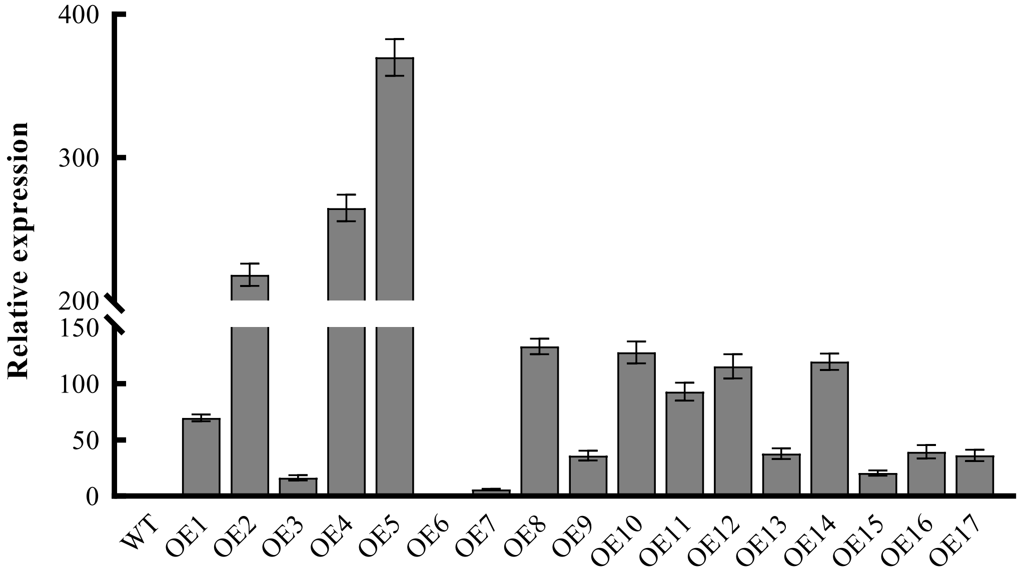

Supplementary Figure S5 Relative expression level of pCAMBIA1305.1-35S-*BnaC09.OGT*-NOS independent T_0 transgenic lines. The values are the means ± SDs, *n* = 3; WT: untransformed 'Zhongshuang 11'. OE1-OE17: independent line of pCAMBIA1305.1-35S-*BnaC09.OGT*-NOS transformants in 'Zhongshuang 11' genetic background.

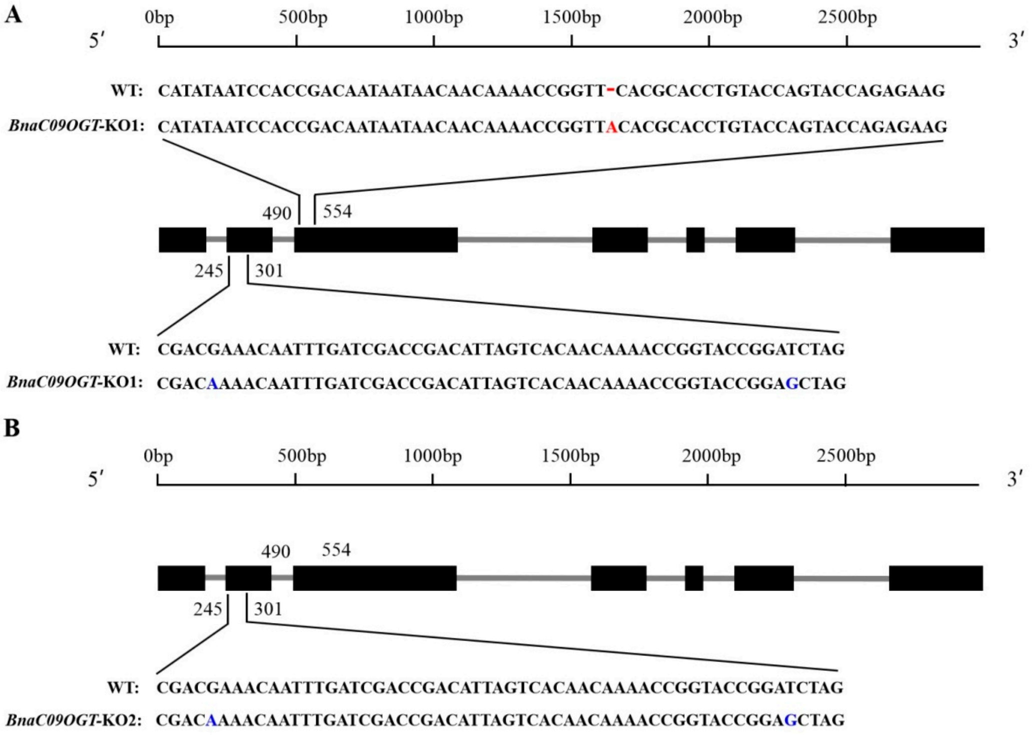

Supplementary Figure S6 Sequence characteristics of mutation sites in T_0 generation *BnaC09.OGT* edited lines. (A) The mutation site in Bna*C09.OGT*-CR1, (B) The mutation site in *BnaC09.OGT*-CR2. Blue and red characters indicate transition/transversion and insertion, respectively.
